# Stereotactic Radiosurgery for Contrast-Enhancing Satellite Nodules in Recurrent Glioblastoma: A Rare Case Series From a Single Institution

**DOI:** 10.7759/cureus.44455

**Published:** 2023-08-31

**Authors:** David J Park, Amit R Persad, Kelly H Yoo, Neelan J Marianayagam, Ulas Yener, Armine Tayag, Louisa Ustrzynski, Sara C Emrich, Cynthia Chuang, Erqi Pollom, Scott G Soltys, Antonio Meola, Steven D Chang

**Affiliations:** 1 Department of Neurosurgery, Stanford University School of Medicine, Stanford, USA; 2 Department of Radiation Oncology, Stanford University School of Medicine, Stanford, USA

**Keywords:** o6-methylguanine-dna methyltransferase, isocitrate dehydrogenase, multicentric, multifocal, satellite, recurrent, radiosurgery, glioblastoma

## Abstract

Introduction

Glioblastoma (GBM) is the most common malignant adult brain tumor and is invariably fatal. The standard treatment for GBM involves resection where possible, followed by chemoradiation per Stupp's protocol. We frequently use stereotactic radiosurgery (SRS) as a single-fraction treatment for small (volume ≤ 1cc) nodular recurrent GBM to the contrast-enhancing target on T1 MRI scan. In this paper, we aimed to evaluate the safety and efficacy of SRS for patients with contrast-enhancing satellite nodules in recurrent GBM.

Methods

This retrospective study analyzed the clinical and radiological outcomes of five patients who underwent CyberKnife (Accuray Inc., Sunnyvale, California) SRS at the institute between 2013 and 2022.

Results

From 96 patients receiving SRS for GBM, five (four males, one female; median age 53) had nine distinct new satellite lesions on MRI, separate from their primary tumor beds. Those nine lesions were treated with a median margin dose of 20 Gy in a single fraction. The three-, six, and 12-month local tumor control rates were 77.8%, 66.7%, and 26.7%, respectively. Median progression-free survival (PFS) was seven months, median overall survival following SRS was 10 months, and median overall survival (OS) was 35 months. Interestingly, the only lesion that did not show radiological progression was separate from the T2-fluid attenuated inversion recovery (FLAIR) signal of the main tumor.

Conclusion

Our SRS treatment outcomes for recurrent GBM satellite lesions are consistent with existing findings. However, in a unique case, a satellite nodule distinct from the primary tumor's T2-FLAIR signal and treated with an enlarged target volume showed promising control until the patient's demise. This observation suggests potential research avenues, given the limited strategies for 'multicentric' GBM lesions.

## Introduction

Glioblastoma (GBM) is the most common primary malignant brain tumor and is invariably fatal in adults [1]. Based on the updated 2021 WHO guidelines, the nomenclature GBM is used to refer to isocitrate dehydrogenase (IDH) wild-type tumors only [2, 3]. Despite multimodality treatment, including surgery, radiation therapy, and chemotherapy, the median survival rate remains only 12-18 months [4]. Thus, there is a need for innovative approaches to treat this disease [5].

One potential alternative treatment modality for recurrent GBM is stereotactic radiosurgery (SRS), which offers a minimally invasive option that could potentially prolong survival [6-15]. However, evidence supporting the use of SRS in recurrent GBM is inconclusive. A retrospective observational international multicenter study focusing on SRS for GBM demonstrated a median progression-free survival (PFS) of four months following SRS treatment, with a median overall survival (OS) of eight months after SRS and 26 months after the initial diagnosis [16].

SRS might be an attractive option for focal recurrences, especially given its submillimeter accuracy and steep dose gradient. In particular, small contrast-enhancing lesions separate from the primary tumor (satellite lesions) in recurrent GBM might be ideal targets for SRS. In such cases, surgical resection may not be feasible. Anecdotally, SRS has been used to treat such lesions in a single fraction analogous to metastatic brain lesions, although its effectiveness has not been previously studied. A major difficulty in using SRS for GBM is determining what should be targeted for treatment, which, in this study, we define as the new contrast-enhancing nodules only.

The purpose of this study is to evaluate the safety and effectiveness of SRS in treating patients with small areas of nodular enhancement in recurrent GBM separate from the primary tumor.

This article was previously posted to the ResearchSquare preprint server on April 14, 2023.

## Materials and methods

We retrospectively reviewed the IRB-approved institutional adult brain tumor database between January 2013 and December 2022. The collected clinical information adheres to the principles of the Helsinki Declaration. Given that our study focused on retrospective evaluation of already-existing, fully anonymized medical records, with no potential to retrace back to individual patients, it was determined that patient consent was unnecessary and, thus, was not pursued.

In this study, our primary interest was in patients diagnosed with recurrent GBM exhibiting small nodular foci of enhancement who underwent CyberKnife (CK; Accuray Inc., Sunnyvale, California) SRS at our facility. For the purpose of this study, GBM was defined according to the WHO 2021 guidelines, exclusively considering tumors that were IDH wild-type.

For this study, we established specific criteria. Our inclusion criteria were as follows: 1) we considered patients diagnosed with recurrent GBM, specifically those presenting small nodular enhancements distant from the contrast-enhancing region of their initial tumor bed; 2) we included individuals who had undergone CK SRS treatment at our facility; 3) we restricted our scope to tumors that align with the IDH wild-type as per the WHO 2021 guidelines; and 4) we categorized lesions as 'small' if their T1-contrast enhancing volume was less than 1cc.

On the other hand, our exclusion criteria included: 1) patients who exhibited a GBM recurrence precisely at the site of the initial resection cavity and 2) cases where the contrast-enhancing nodules were contiguous with the contrast enhancement stemming from the initial tumor bed.

Our analysis emphasized both the clinical and radiological outcomes of the aforementioned patient group.

Stereotactic radiosurgery technique

The study used the CyberKnife technique to treat patients with distant small recurrent IDH-wt GBM. All patients received CK treatment as previously described [17].

Statistical analysis

We computed overall survival (OS), progression-free survival (PFS), and local tumor control (LTC) rates using data from the initial diagnosis, date of SRS, follow-up MRI studies, and last follow-up or death. Categorical variables were evaluated through frequencies and percentages, and means, medians, and ranges were provided for continuous variables. Subgroup analysis was not performed due to the limited size of the cohort. The statistical analysis was done using standard statistical processing software (SPSS statistics version 28.0; IBM Inc, Armonk, New York).

## Results

Patient demographics and clinical data

Of 96 patients undergoing SRS for GBM, we identified five patients with histopathologically confirmed GBM who underwent a single-fraction SRS for a total of nine new small contrast-enhancing satellite lesions seen on MRI (Table 1). These lesions were located separately from the primary lesion, which remained stable at the time of SRS treatment. The patients included four males and one female with a median age of 53 years (range 24-63). All patients underwent craniotomy and resection of the tumor at the primary site, which was pathologically confirmed as GBM with wild-type IDH status. The O6-methylguanine-DNA methyltransferase (MGMT) methylation status was positive for three patients and negative for two patients. All patients underwent standard chemoradiation with temozolomide (TMZ) and fractionated radiotherapy [5, 18]. Additional systemic therapy included bevacizumab (Avastin) in four patients and lomustine (CCNU), carmustine (BCNU), and irinotecan for one patient prior to SRS treatment (Table 1). Supplementary radiation to the primary lesion included CK SRS to the resection cavity with 2 mm margins, which was applied in two patients (Table 1). Patient follow-up was carried out in the clinic as well as by EMR reports.

**Table 1:** Patient demographics and clinical profiles PFS - progression-free survival; GTV - Gross tumor volume; PTV - Planning target volume; FLAIR - T2-weighted fluid-attenuated inversion recovery; Dmax - maximum dose; BED - biologically effective dose; RT - radiation therapy; SRS - stereotactic radiosurgery; RC - resection cavity; TTF - tumor treating field; IDH - isocitrate dehydrogenase; MGMT - O6-methylguanine-DNA methyltransferase; TMZ - temozolomide; CCNU - lomustine; BCNU - carmustine; m - months

Age	Sex	Location	GTV (cc)	PTV (cc)	FLAIR Connection	Dmax (Gy)	Margin dose (Gy)	Isodose line (%)	BED (Gy)	Surgical resection	Prior RT	Prior chemotherapy	Other treatment	IDH mutation	MGMT methylation	Follow-up duration (m)	PFS (m)	OS after SRS (m)	OS (m)
56	F	Left lateral temporal	0.32	0.32	Y	28.08	20	71	70	Y	Y	TMZ, bevacizumab	N	N	P	18	14	18	52
		Left medial temporal	0.16	0.16	Y	28.17	20	71	70								14		
63	F	Left posterior temporal	0.91	2.56	Y	31.42	22	70	82.5	Y	Y	TMZ, bevacizumab	SRS to RC, TTF	N	P	5	2	5	48
		Left medial occipital	0.57	3.67	Y	31.42	22	70	82.5								2		
		Left anterior temporal	0.76	3.98	N	31.42	22	70	82.5								-		
53	M	Corpus callosum	0.8	0.8	Y	25.35	18	71	58.5	Y	Y	TMZ, BCNU, irinotecan	N	N	P	7	7	7	35
		Left inferior frontal	1	1	Y	25	18	72	58.5								7		
24	F	Right frontal	0.98	0.98	Y	22.5	18	80	58.5	Y	Y	TMZ, bevacizumab	N	N	N	10	4	10	17
39	F	Right posterior temporal	0.03	0.43	Y	32	24	75	96	Y	Y	TMZ, bevacizumab, CCNU	SRS to RC	N	N	12	9	12	18

Stereotactic radiosurgery characteristics

**Figure 1:**
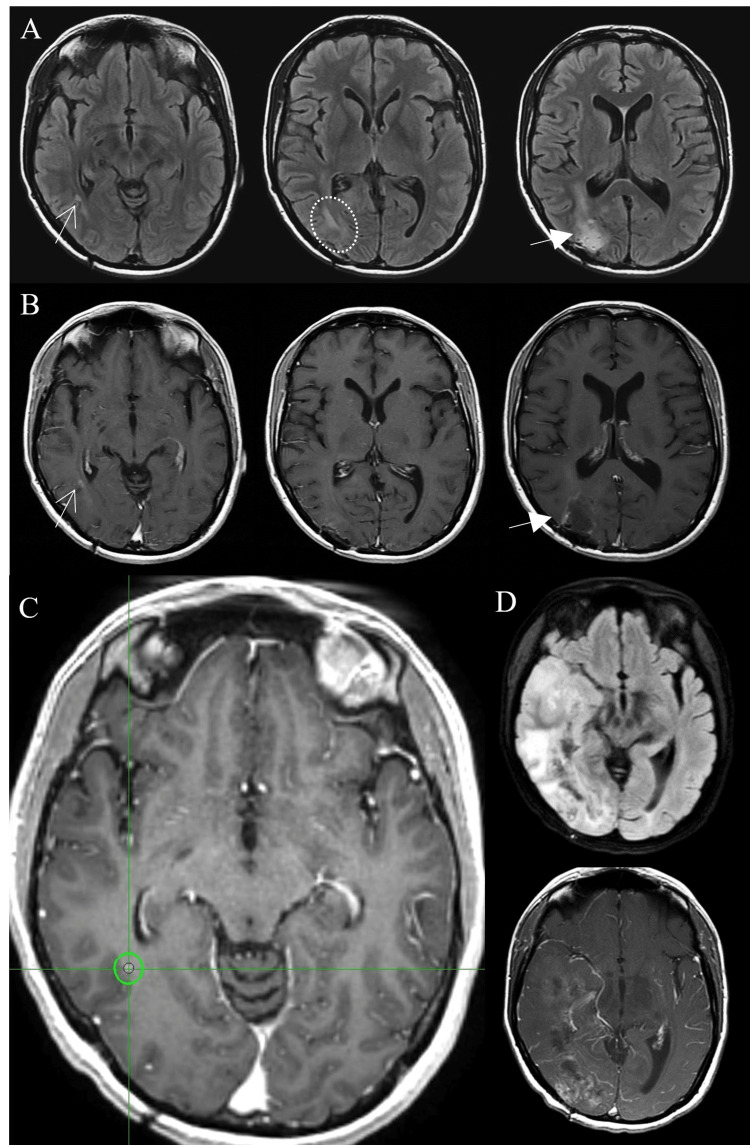
Satellite lesion with T2-FLAIR connection to the primary lesion. A and B) MRI studies before stereotactic radiosurgery (SRS) treatment (A: FLAIR, B: T1 with contrast). Arrows with lined tip indicates the satellite lesion, and the arrows with triangular tip indicates the surgical cavity of the primary GBM. Circular dotted area demonstrates the T2-FLAIR connection between those two lesions, distant recurrence and primary site. C) SRS plan for the recurrent nodular GBM. The gross tumor volume (GTV, contoured in red), a volume to T1-contrast enhancing area, was 0.03 cc and the planning target volume (PTV, contoured in green) was 0.43 cc. A marginal dose of 24 Gy, with a maximum dose of 32 Gy, was delivered in a single fraction to 75% isodose line. D) MRI study of nine-month follow-up demonstrates the progression of GBM from both the primary site and satellite lesion where SRS was delivered (upper: FLAIR, lower: T1 with contrast).

**Figure 2:**
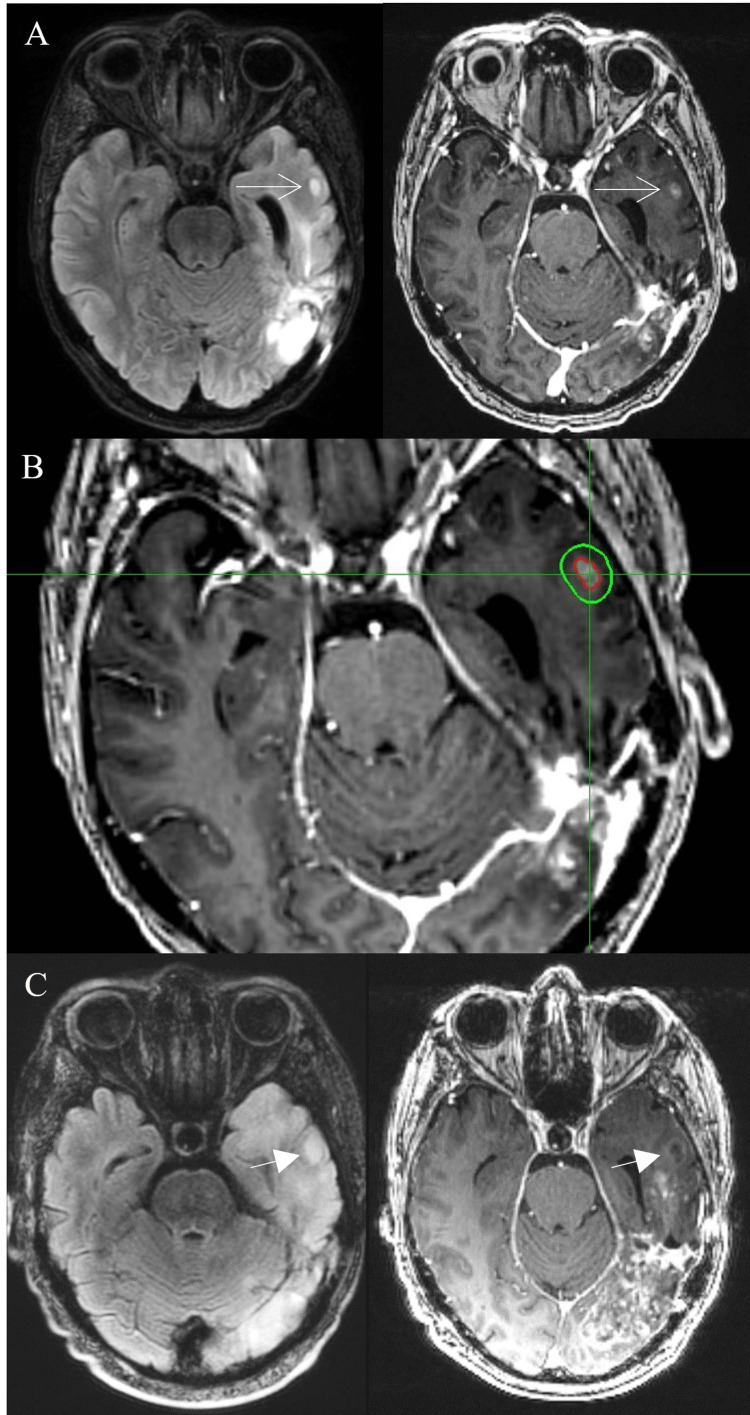
Satellite lesion with no T2-FLAIR connection to the primary lesion. A) MRI studies before CyberKnife radiosurgery treatment (left: FLAIR, right: T1 with contrast). Arrows with lined tips indicate the satellite lesion in the left temporal area, which is not connected with T2-FLAIR hyperintensity from the primary site in the left occipital area. B) CyberKnife radiosurgery plan for the recurrent nodular glioblastoma (GBM). The gross tumor volume (GTV, contoured in red), a volume to T1-contrast enhancing area, was 0.76 cc and the planning target volume (PTV, contoured in green) was 3.98 cc. A marginal dose of 22 Gy, with a maximum dose of 31.41 Gy, was delivered in a single fraction to 70% isodose line. C: MRI study of five-month follow-up demonstrates no progression of GBM at the satellite lesion where SRS was delivered (left: FLAIR, right: T1 with contrast). The size of the lesion is stable, and the contrast enhancement in the core portion has disappeared. However, due to the progression of the primary site in the left occipital area, which is revealed in T1-contrast MRI (right), the patient died having an overall survival of 48 months since the initial diagnosis.

CK treatment took place after a median duration of 27 months (range 6-42 months) from the initial diagnosis. The patients had a median of two small lesions (range 1-3) treated with CK SRS. CK plans of two representative cases are presented in Figures 1 and 2. The median gross tumor volume (GTV) was 0.57 cc (range 0.03-0.98). Five of the nine lesions were treated with CK based on GTV without any volumetric expansion. For the other four lesions, the planning target volume (PTV) was contoured considering T2-FLAIR hyperintense area surrounding the T1 contrast-enhancing lesion in the MRI (Table 1). The volume expansion from GTV to PTV ranged from 1.65 to 4.03 cc, which correlates from 181 to 1433% of the GTV. As a result, the median PTV for all nine lesions was 0.98 cc (range 0.16-3.98). The tumor volume was measured using the Accuray Precision® CyberKnife treatment planning system. All SRS treatments were delivered in a single fraction, with a median dose of 20 Gy (range 18-24) to a median isodose line of 71% (range 70-80). Details of the SRS plans are summarized in Table 1.

Radiosurgical outcomes

Imaging follow-up studies demonstrated LTC rates of 77.8%, 66.7%, and 26.7% at three, six, and 12 months, respectively, after SRS. The median PFS for the SRS-treated small lesion was seven months. The median OS for all five patients after SRS was 10 months (range 5-18), and the median OS from the initial diagnosis until death was 35 months (range 17-52) (Table 1). Based on the observation that no lesions regressed post-SRS without treatment changes, we found no evidence of pseudoprogression. All five patients tolerated the CyberKnife SRS treatment well without complications, and there were no radiation-induced adverse events.

Only one lesion, in patient two (Table 1), did not reveal progression for five months until the patient passed away due to progression of the primary lesion (Figure 2). All other patients demonstrated radiographic progression within a median of seven months (range 2-14) from the date of SRS treatment.

Interestingly, the only lesion that exhibited no radiological progression was not connected to or encompassed by the T2-FLAIR hyperintense region adjacent to the primary tumor bed on the MRI. In this case, the PTV was extended 2-3mm beyond the GTV boundary (which was defined by the T1-contrast enhancing area). The PTV encompassed much of the T2-FLAIR signal surrounding the enhancing nodule.

## Discussion

In this study, we explored the efficacy of SRS for nodular foci of contrast enhancement separate from the primary lesion in recurrent glioblastoma. Despite the use of multimodal treatments, including surgery, radiation, and chemotherapy, the prognosis for GBM remains bleak. In most cases, GBM recurs near the initial tumor site [19]. Various treatment approaches have been explored, such as surgery, SRS, target therapy, immunotherapy, and combinations of these, producing diverse survival rates [6]. Nonetheless, no substantial breakthrough in standard GBM treatment has occurred since the Stupp protocol. Previously, external beam radiotherapy (EBRT) was the primary radiation treatment modality for GBM, but as radiation technology has improved, the treatment target has progressively become more focused on the T1-contrast enhancing area without significantly affecting patient prognoses. Tumor edge definition and target delineation are essential components of radiation therapy, and many studies have shown that treatment failures for GBM are more likely within 2-3 cm of the primary tumor [20, 21]. The Radiation Therapy Oncology Group (RTOG) [22] and the European Organization for Research and Treatment of Cancer (EORTC) [23] have published widely used radiotherapy protocols for GBM, and there are several other guidelines related to radiotherapy for GBM [24, 25]. However, all studies on target delineation in GBM are based on radiotherapy.

Currently, there is no consensus on the target delineation for SRS treatment, specifically regarding how much of the T2-FLAIR signal should be included. The T2-FLAIR signal corresponds to non-enhancing tumor and to peritumoral edema. Theoretical arguments both for and against the inclusion of the T2-FLAIR signal as a target volume exist [26, 27]. The theoretical basis for including the T2-FLAIR signal is for more comprehensive inclusion of tumor burden [26-29]. Conceptually, this is similar to supramarginal resection during surgery [27]. One prior study examined this concept with promising preliminary results [30]. Duma et al. defined leading-edge radiosurgery as targeting white matter pathways both adjacent to and leading away from the original contrast-enhancing tumor site [30]. The patients who underwent leading-edge radiosurgery for GBM had a median overall survival of 23 months, with some survival rates notably extending up to 10 years after treatment [30]. The theoretical rationale for excluding the T2-FLAIR signal is that recurrence patterns are similar regardless of inclusion [31, 32]. Additionally, including the T2-FLAIR signal in the target volume raises the radiation volume of normal brain tissue and may increase the risk of brain injury [32], although some studies show no increased adverse event profile [33].

A number of approaches utilizing SRS for GBM have been studied and evaluated. Prior research has reported the T1-contrast enhancing lesion as the primary target for SRS treatment, with only a few cases targeting the T2-FLAIR hyperintensity [30]. Our group conducted a phase I/II trial of five-fraction SRS with 5 mm margins with concurrent temozolomide chemotherapy in newly diagnosed glioblastoma, with a 30-patient cohort demonstrating median PFS and OS of 8.2 months and 14.8 months, respectively [8]. Retrospective multi-institutional analysis of SRS for recurrent GBM in a 46-patient cohort reported a median OS of nine months after SRS and 23.8 months after diagnosis [6], while a retrospective observational international multicenter study of SRS for IDH-wt GBM showed a median PFS of four months after SRS treatment and a median OS of eight and 26-month after SRS and initial diagnosis, respectively [16]. Based on prior reports, the median PFS after SRS for GBM ranged from 3.4 months to 14.9 months, and the median OS after SRS and diagnosis ranged from 5.3 to 17.9 months and 16.7 to 33.2 months, respectively [10, 34]. In our case series, the median PFS was 10 months after SRS, while the median OS was seven months after SRS and 35 months after diagnosis, which was consistent with previous research. The LTC at six months in our series is 66.7%, which is similar to a previous series reporting 68% [7].

Several prognostic factors have been identified as related to improved survival following SRS treatment for GBM. These include smaller tumor volumes (<5 cc, <14 cc, <15cc) [7, 10, 16, 34, 35] and higher SRS prescription doses (>14 Gy or >15 Gy) [6, 7, 10, 16], which appear to be related to tumor volume. Other factors include younger age at diagnosis (<60 years or <50 years) [6, 10, 35], higher Karnofsky Performance Scale score (≥ 80) [33], prior gross-total resection [7], use of prior chemotherapy [35], and radiosurgery at the time of recurrence [35]. However, IDH mutation and MGMT promoter methylation status were not found to be statistically significant for post-SRS survival [7]. Based on the literature, SRS might be most reasonable as a treatment for small nodular foci of GBM with higher SRS prescription doses [6, 7, 16].

Recently updated evidence-based clinical practice guidelines offered insights into the role of diverse radiation therapy in the management of recurrent and progressive GBM in adults [36]. The guidelines identified nine sources of level III evidence. Although the literature reports varied endpoints and outcome parameters, level III evidence suggests that for adult patients with progressive/recurrent glioblastoma multiforme after first-line combined multimodality treatment, re-irradiation can enhance tumor control, prolong PFS, and better neurological and functional outcomes [36]. Such re-irradiation includes various forms, such as conventional fractionation radiotherapy, fractionated radiosurgery, or single fraction radiosurgery. While previous studies have focused on the efficacy of radiation treatment on GBM at the original site [6-8, 10, 16, 37], our case series focuses on small lesions with T1-contrast enhancing satellite lesions.

In our case series, the survival outcomes aligned with those from other studies. Overall, our findings are in agreement with the results of SRS for recurrent GBM found in other research. However, while our objective was to target small contrast-enhancing satellite lesions with higher SRS prescription doses in hopes of markedly improved outcomes, including local tumor control, PFS, and OS, the results did not meet our anticipated standards.

Out of a total of nine lesions, only one lesion did not show clear progression until the patient's death. Two factors distinguished that particular lesion from the others. Firstly, it was the sole lesion located outside of the T2-FLAIR signal surrounding the primary tumor. Secondly, the lesion was treated with a volume-expanded PTV encompassing its own T2-FLAIR signal. Taken together, such observations might imply that SRS to GBM lesions encompassing their T2-FLAIR signal is a valid strategy for tumor control. Such an approach might be effective for satellite lesions that are separate from the T2-FLAIR signal of primary tumors. In terms of pathophysiology, the distinction could be due to one being a progression of 'multifocal' recurrent GBM, while the latter could represent a 'multicentric' GBM lesion. Multicentric GBM is not linked by the T2-FLAIR signal and may carry worse outcomes. Treatment strategies for multicentric GBM are lacking, and our data may offer a preliminary indication that SRS could be considered for such lesions.

Limitations

There are several significant limitations to our study that must be acknowledged. Due to its retrospective design and small cohort size, our study cannot draw definitive conclusions. Moreover, statistical analysis for this paper was not performed, as the limited number of patients would prevent any calculation of significance. We only selected patients who underwent molecular assessment of IDH status, which has only become ubiquitously tested as a standard of care in diagnosis within the last decade. The diverse treatment plans for recurrent lesions limit the ability to make comparisons. Furthermore, due to the retrospective design, we were unable to investigate diverse and personalized chemotherapy regimens that could impact outcomes. As we focused only on assessing the safety and efficacy of SRS for small satellite lesions in recurrent GBM, we did not have a control cohort of patients treated with other modalities at disease recurrence.

Consequently, our findings may only apply to this particular small cohort and do not demonstrate a comparison with alternative treatment regimens beyond the scope of this study. In addition, the SRS target definition was not standardized even within our single institution over time. We treated all patients using the CyberKnife SRS device with a single fraction only, and thus, our results should be extrapolated with caution to other SRS treatment systems and different radiotherapy fractionation schemes.

Despite these limitations, this small case series from a single institution may provide a starting point for further studies. Our data suggests that SRS to contrast-enhancing satellite nodules in recurrent GBM does not improve tumor control or survival. In particular, we would encourage further studies on SRS treatment to the T2-FLAIR signal and the utility of SRS in multicentric GBM.

## Conclusions

In our study, the survival outcomes of SRS treatment for recurrent GBM satellite lesions mirrored those from existing studies. While our intent was to enhance outcomes for small contrast-enhancing satellite lesions with higher SRS doses, the anticipated results were not universally observed. Nevertheless, out of the nine lesions examined, we noted a distinct case where a satellite nodule, separate from the primary tumor's T2-FLAIR signal and treated with expanded target volume, showed effective local tumor control until the patient's demise. This singular outcome suggests potential avenues for further research, particularly considering the dearth of strategies targeting 'multicentric' GBM lesions.

## References

[1] Ostrom QT, Price M, Neff C, Cioffi G, Waite KA, Kruchko C, Barnholtz-Sloan JS (2022). CBTRUS statistical report: Primary brain and other central nervous system tumors diagnosed in the United States in 2015-2019. Neuro Oncol.

[2] Stoyanov GS, Lyutfi E, Georgieva R (2022). Reclassification of glioblastoma multiforme according to the 2021 World Health Organization classification of central nervous system tumors: a single institution report and practical significance. Cureus.

[3] Antonelli M, Poliani PL (2022). Adult type diffuse gliomas in the new 2021 WHO Classification. Pathologica.

[4] Tan AC, Ashley DM, López GY, Malinzak M, Friedman HS, Khasraw M (2020). Management of glioblastoma: state of the art and future directions. CA Cancer J Clin.

[5] Stupp R, Mason WP, van den Bent MJ (2005). Radiotherapy plus concomitant and adjuvant temozolomide for glioblastoma. N Engl J Med.

[6] Lovo EE, Moreira A, Barahona KC (2021). Stereotactic radiosurgery for recurrent glioblastoma multiforme: a retrospective multi-institutional experience. Cureus.

[7] Bunevicius A, Pikis S, Kondziolka D (2021). Stereotactic radiosurgery for glioblastoma considering tumor genetic profiles: an international multicenter study. J Neurosurg.

[8] Azoulay M, Chang SD, Gibbs IC (2020). A phase I/II trial of 5-fraction stereotactic radiosurgery with 5-mm margins with concurrent temozolomide in newly diagnosed glioblastoma: primary outcomes. Neuro Oncol.

[9] Morris SL, Zhu P, Rao M (2019). Gamma Knife stereotactic radiosurgery in combination with bevacizumab for recurrent glioblastoma. World Neurosurg.

[10] Niranjan A, Monaco EA II, Kano H, Flickinger JC, Lunsford LD (2018). Stereotactic radiosurgery in the multimodality management of residual or recurrent glioblastoma multiforme. Prog Neurol Surg.

[11] Holt DE, Bernard ME, Quan K, Clump DA, Engh JA, Burton SA, Heron DE (2016). Salvage stereotactic radiosurgery for recurrent glioblastoma multiforme with prior radiation therapy. J Cancer Res Ther.

[12] Redmond KJ, Mehta M (2015). Stereotactic radiosurgery for glioblastoma. Cureus.

[13] Nwokedi EC, DiBiase SJ, Jabbour S, Herman J, Amin P, Chin LS (2002). Gamma knife stereotactic radiosurgery for patients with glioblastoma multiforme. Neurosurgery.

[14] Cho KH, Hall WA, Lo SS, Dusenbery KE (2004). Stereotactic radiosurgery versus fractionated stereotactic radiotherapy boost for patients with glioblastoma multiforme. Technol Cancer Res Treat.

[15] Biswas T, Okunieff P, Schell MC (2009). Stereotactic radiosurgery for glioblastoma: retrospective analysis. Radiat Oncol.

[16] Bunevicius A, Pikis S, Kondziolka D (2021). Stereotactic radiosurgery for IDH wild type glioblastoma: an international, multicenter study. J Neurooncol.

[17] Gibbs IC (2006). Frameless image-guided intracranial and extracranial radiosurgery using the Cyberknife robotic system. Cancer Radiother.

[18] Erpolat OP, Akmansu M, Goksel F, Bora H, Yaman E, Büyükberber S (2009). Outcome of newly diagnosed glioblastoma patients treated by radiotherapy plus concomitant and adjuvant temozolomide: a long-term analysis. Tumori.

[19] Birzu C, French P, Caccese M, Cerretti G, Idbaih A, Zagonel V, Lombardi G (2020). Recurrent glioblastoma: From molecular landscape to new treatment perspectives. Cancers (Basel).

[20] Wallner KE, Galicich JH, Krol G (1989). Patterns of failure following treatment for glioblastoma multiforme and anaplastic astrocytoma. Int J Radiat Oncol Biol Phys.

[21] Nie S, Zhu Y, Yang J (2021). Clinicopathologic analysis of microscopic tumor extension in glioma for external beam radiotherapy planning. BMC Med.

[22] Sulman EP, Ismaila N, Armstrong TS (2017). Radiation therapy for glioblastoma: American Society of Clinical Oncology clinical practice guideline endorsement of the American Society for Radiation Oncology guideline. J Clin Oncol.

[23] Niyazi M, Brada M, Chalmers AJ (2016). ESTRO-ACROP guideline "target delineation of glioblastomas". Radiother Oncol.

[24] Tseng CL, Stewart J, Whitfield G (2020). Glioma consensus contouring recommendations from a MR-Linac International Consortium Research Group and evaluation of a CT-MRI and MRI-only workflow. J Neurooncol.

[25] Kruser TJ, Bosch WR, Badiyan SN (2019). NRG brain tumor specialists consensus guidelines for glioblastoma contouring. J Neurooncol.

[26] Li M, Huang W, Chen H (2022). T2/FLAIR abnormity could be the sign of glioblastoma dissemination. Front Neurol.

[27] Haddad AF, Young JS, Morshed RA, Berger MS (2022). FLAIRectomy: Resecting beyond the contrast margin for glioblastoma. Brain Sci.

[28] Schoenegger K, Oberndorfer S, Wuschitz B (2009). Peritumoral edema on MRI at initial diagnosis: an independent prognostic factor for glioblastoma?. Eur J Neurol.

[29] Yamahara T, Numa Y, Oishi T, Kawaguchi T, Seno T, Asai A, Kawamoto K (2010). Morphological and flow cytometric analysis of cell infiltration in glioblastoma: a comparison of autopsy brain and neuroimaging. Brain Tumor Pathol.

[30] Duma CM, Kim BS, Chen PV (2016). Upfront boost Gamma Knife "leading-edge" radiosurgery to FLAIR MRI-defined tumor migration pathways in 174 patients with glioblastoma multiforme: a 15-year assessment of a novel therapy. J Neurosurg.

[31] Chang EL, Akyurek S, Avalos T (2007). Evaluation of peritumoral edema in the delineation of radiotherapy clinical target volumes for glioblastoma. Int J Radiat Oncol Biol Phys.

[32] Minniti G, Amelio D, Amichetti M (2010). Patterns of failure and comparison of different target volume delineations in patients with glioblastoma treated with conformal radiotherapy plus concomitant and adjuvant temozolomide. Radiother Oncol.

[33] Choi SH, Kim JW, Chang JS, Cho JH, Kim SH, Chang JH, Suh CO (2017). Impact of including peritumoral edema in radiotherapy target volume on patterns of failure in glioblastoma following temozolomide-based chemoradiotherapy. Sci Rep.

[34] Sharma M, Schroeder JL, Elson P (2018). Outcomes and prognostic stratification of patients with recurrent glioblastoma treated with salvage stereotactic radiosurgery. J Neurosurg.

[35] Niranjan A, Kano H, Iyer A, Kondziolka D, Flickinger JC, Lunsford LD (2015). Role of adjuvant or salvage radiosurgery in the management of unresected residual or progressive glioblastoma multiforme in the pre-bevacizumab era. J Neurosurg.

[36] Ziu M, Goyal S, Olson JJ (2022). Congress of Neurological Surgeons systematic review and evidence-based guidelines update on the role of radiation therapy in the management of progressive and recurrent glioblastoma in adults. J Neurooncol.

[37] Shah JL, Li G, Shaffer JL, Azoulay MI, Gibbs IC, Nagpal S, Soltys SG (2018). Stereotactic radiosurgery and hypofractionated radiotherapy for glioblastoma. Neurosurgery.

